# Biology of Pellino1: a potential therapeutic target for inflammation in diseases and cancers

**DOI:** 10.3389/fimmu.2023.1292022

**Published:** 2023-12-18

**Authors:** Lili Yan, Yueran Cui, Juan Feng

**Affiliations:** Department of Neurology, Shengjing Hospital of China Medical University, Shenyang, China

**Keywords:** Peli1, ubiquitination, IL-1R/TLRs, NF-κB, inflammation, cancer, immune

## Abstract

Pellino1 (Peli1) is a highly conserved E3 Ub ligase that exerts its biological functions by mediating target protein ubiquitination. Extensive evidence has demonstrated the crucial role of Peli1 in regulating inflammation by modulating various receptor signaling pathways, including interleukin-1 receptors, Toll-like receptors, nuclear factor−κB, mitogen-activated protein kinase, and phosphoinositide 3-kinase/AKT pathways. Peli1 has been implicated in the development of several diseases by influencing inflammation, apoptosis, necrosis, pyroptosis, autophagy, DNA damage repair, and glycolysis. Peli1 is a risk factor for most cancers, including breast cancer, lung cancer, and lymphoma. Conversely, Peli1 protects against herpes simplex virus infection, systemic lupus erythematosus, esophageal cancer, and toxic epidermolysis bullosa. Therefore, Peli1 is a potential therapeutic target that warrants further investigation. This comprehensive review summarizes the target proteins of Peli1, delineates their involvement in major signaling pathways and biological processes, explores their role in diseases, and discusses the potential clinical applications of Peli1-targeted therapy, highlighting the therapeutic prospects of Peli1 in various diseases.

## 1 Introduction

Ubiquitin (Ub) is an evolutionarily conserved polypeptide that plays a crucial role in post-translational modifications by binding to protein substrates, thus regulating signaling in various receptor systems (1, 2). Ubiquitination involves several reactions mediated by three classes of ubiquitin-related enzymes. First, the ubiquitin-activating enzyme (E1) activates the Ub molecules. Subsequently, the Ub-conjugating enzyme (E2) receives an activated Ub molecule from E1. Lastly, the Ub-ligase (E3) transfers the Ub molecule bound to E2 to the target protein (1).

Pellino1 (Peli1) has been extensively studied among the E3 Ub ligase family members. Initially identified in Drosophila, Peli1 has a molecular mass of 47 kDa and interacts with Pelle, a homolog of interleukin-1 receptor-associated kinase 1 (IRAK1) (3). Three members of the mammalian Peli family (Peli1, Peli2, and Peli3) have been identified, along with two selectively spliced forms of Peli3, namely Pellino3a and Pellino3b (4, 5). The N-terminal region of Peli contains a core forkhead-associated (FHA) structural domain, characterized by two inserted fragments forming a “wing” or appendage tightly bound to the FHA domain (6). Similar to the classical C_3_HC_4_ loop structure, the C-terminus of the Peli family features a RING-like domain encompassing two stable Cys-Gly-His motifs and two conserved CysPro-X-Cys motifs, enabling ubiquitination through K11, K48, and K63 linkages (7, 8). Peli1 phosphorylation is necessary for its E3 Ub- ligase activity. Substrate recognition is facilitated by the FHA structural domain, with the amino acid located at the +3 position relative to the phosphorylated threonine, a key determinant of PT peptide recognition by many FHA domains. Different Pellinos exhibit diverse phosphothreonine peptide binding properties (9). The strong binding of Peli1 to IRAK1 and receptor-interacting protein kinase 1 (RIP1) is associated with its preference for pTxxY- or pTxxS-binding motifs, whereas Pellinos no show a preference for binding to the pT141 + 3D motif (9).

Peli1 modulates immune response, cell death, autophagy, DNA damage repair, and glycolysis through its E3 Ub ligase activity in a dependent or independent manner. Peli1 exhibits remarkable versatility in immune regulation by participating in interleukin-1 receptors (IL-1R), Toll-like receptors (TLRs), nuclear factor−κB (NF-κB), mitogen-activated protein kinase (MAPK), and phosphoinositide 3-kinase (PI3K)/AKT pathways. These multifaceted pathways involving Peli1 regulate numerous diseases, particularly tumors, inflammatory disorders, and autoimmune conditions. E3 ligase-related targeted regulation is common; for instance, the E3 ligase SKP1-CUL1-F-box-protein (SCF) or Cullin-RING E3 ligase (CRL) has been investigated with several targeted inhibitors and agonists. For example, inhibitors such as compound A blocks substrate SKP2 binding to the SCF complex, and agonists, such as thalidomide, primarily target cereblon (CRBN), a substrate-recognizing subunit of the CUL4-DDB1 E3 Ub ligase complex (10, 11). The substrates regulated by Peli1 as an E3 ligase are also relatively specific (**Table 1**); therefore, selectively targeting Peli1 is essential. A few strategies to target and manipulate Peli1 for therapeutic benefits, underscoring its potential as a therapeutic target, such as the inhibitors BBT-401 and S62 (22, 48).

**Table 1:** Proteins targeted by Peli1-mediated ubiquitination or ubiquitination-independent.

Interaction	Target Protein	Modifying site	Association	Ref
K48	RIP3	K363	Toxic epidermal necrolysis (TEN)	(12)
K48	c-Rel		T cell activation	(13)
K48,K63	BubR1		Tumor	(14)
K48	NIK		SLE, Esophageal squamous cancer	(15, 16)
K48	HPD		Tyrosinemia	(17)
K48	LAMP2		Parkinson’s disease	(18)
K48	C/EBPβ		Alzheimer’s disease (AD)	(19)
K48	PKCθ		Tumor	(20)
K48	LSD1		Breast cancer	(21)
K63	EGFR		Breast cancer	(22)
K63	SNAIL/SLUG		Breast cancer, Lung cancer	(23, 24)
K63	cIAP2		CNS inflammation, Endotoxin tolerance, Lung cancer	(25, 26)
K63	RIP1	K115	TLRs signal, Necroptosis	(27, 28)
K63	TRAF6		Morphine Tolerance	(29)
K63	BCL6		Lymphoma	(30)
K63	IRF5		Glucose Intolerance, myocardial Ischemia/reperfusion injury	(31, 32)
K63	MDMX		Tumor, DNA damage response	(33, 34)
K63	NBS1	K686, K690	DNA damage response	(35)
K63	TBK1		TLRs signal	(36)
K63	IRAK1		Tumor	(37)
K63	BECN1		HIV infection	(38)
K63	TSC1	K30,K632	Tumor	(39)
K63	ASC	K55	Inflammasome activation	(40)
K63	P62	K7	Myocardial ischemia/reperfusion injury	(41)
K63	P21		Chronic Obstructive Pulmonary Disease	(42)
E3 Ub ligase activity independent	DEAF1		Sendai Virus infection.	(43)
E3 Ub ligase activity independent	Smad6,Smad7		IL-1R/TLRs	(44, 45)
E3 Ub ligase activity independent	γH2AX		DNA damage response	(33)
E3 Ub ligase activity independent	P53		DNA damage response	(33)
E3 Ub ligase activity independent	IRAK4		DNA damage response	(46)
E3 Ub ligase activity independent	HSP90		Cardiac microvascular endothelium injury	(47)

This review comprehensively examines the signaling pathways involving Peli1, elucidates the target proteins under its regulation, explores their contributions to tumors and inflammation, and discusses the potential clinical applications of Peli1 as a therapeutic target. This comprehensive understanding of the multifunctionality of Peli1 in a pathological contexts provides valuable insights for future research and clinical interventions.

## 2 Molecular function

Peli1 exhibits robust E3 Ub ligase activity, predominantly mediating the Lys11, Lys48, and Lys63 linkages (K11, K48, and K63) of Ub (**Table 1**) (8). *In vivo*, Peli1 primarily facilitates the ubiquitination of target proteins via K48 and K63 linkages and promotes the K48-linked ubiquitination of target proteins, leading to their degradation via the Ub-proteasome system. Peli1 mediates K48-linked Ub (K48-Ub) in the HPD, resulting in its degradation and tyrosinemia (17). Peli1 mediates the K63-linked Ub (K63-Ub) of target proteins, thereby enhancing their stability or promoting subcellular localization, and mediates the K63-Ub in murine double minute X (MDMX), facilitating its nuclear export (49). Peli1 can regulate both K48-Ub and K63-Ub concurrently but differentially, where Peli1 mediates K63-Ub more substantially than the K48-Ub of BubR1 (14). Peli1-mediated K63-Ub of target proteins can signal Met1-linked Ub (M1-Ub) through K63/M1-Ub hybrids. Peli1 ubiquitinates RIP1 via K63-Ub in the TLR signaling pathway (27). Peli1 interacts with molecules and function in an E3 ligase-independent manner facilitated by its FHA structure (**Table 1**).

## 3 Peli1 modulation and modifications

### 3.1 Peli1 regulation in transcription

Several proteins act as transcription factors that regulate Peli1 transcription, and various molecules are involved by modulating the promoters or transcription factors (**Table 2**). One such transcription factor is interferon regulatory factor 3 (IRF3), which regulates Peli1 through the Toll-interleukin-1 receptor -domain-containing adaptor-inducing IFN-beta (TRIF)-dependent pathway of TLR3/4, influencing Peli1 expression (36). The activation of TANK-binding kinase 1 (TBK1) and IkappaB kinase epsilon (Ikkϵ) phosphorylates and activates IRF3, leading to transcriptional Peli1 and type I interferon-beta (IFN-β) upregulation. Subsequently, IFN-β further enhances Peli1 transcription via the Janus kinase/signal transducer and activator of the transcription (JAK/STAT) signaling pathway (36). The TRIF-IRF3 signaling pathway, activated by Bid-dependent mechanisms, contributes to Peli1 transcription upon TLR3 and TLR4 activation (44, 66). The glucocorticoid receptor (GR) interacts with β-arrestin-1, maintaining GR stability and regulating the GR-sensitive region of the Peli1 promoter, thereby influencing Peli1 transcription (50).

**Table 2:** Molecules Regulating Peli1.

Molecule	Type	Modifying Type on Peli1	Association	Ref
IRF3	TF	Promote transcription	TLRs signal pathway	(36)
GR	TF	Promote transcription	Glucocorticoids and immunity	(50)
miR-21	MiRNA	Repress post-transcriptional translation	Idiopathic Pulmonary Fibrosis, Pathogenic TH17 Cells, Liver regeneration	(51–53)
miR-124	MiRNA	Repress post-transcriptional translation	Acute Lung Injury	(54)
miR-135b	MiRNA	Repress post-transcriptional translation	Mycobacterium tuberculosis infection	(55)
miR-142a-3p	MiRNA	Repress post-transcriptional translation	Methamphetamine-induced inflammation	(56)
miR-153-3p	MiRNA	Repress post-transcriptional translation	Systemic lupus erythematosus	(57)
miR-155	MiRNA	Repress post-transcriptional translation	Generation and function of Tfh Cells, Methamphetamine-induced inflammation, Japanese Encephalitis Virus	(56, 58, 59)
miR-301a-3p	MiRNA	Repress post-transcriptional translation	Systemic lupus erythematosus	(60)
miR-590-5p	MiRNA	Repress post-transcriptional translation	Intracerebral hemorrhage, Alzheimer’s disease	(61, 62)
IRAK1	Kinases	Phosphorylation on Ser-76, Ser-78, Thr-80, Ser-82, Thr-86, Thr-288 and Ser-293	IL-1R/TLRs signal pathway	(7, 63)
IRAK4	Kinases	Phosphorylation on Ser-76, Ser-78, Thr-80, Ser-82, Thr-86, Thr-288 and Ser-293	IL-1R/TLRs signal pathway	(7, 63)
TBK1/Ikkϵ	Kinases	Phosphorylation on Ser-76, Thr-288, and Ser-293 sites	IL-1R/TLRs signal pathway	(7, 36)
EGFR	Kinases	Phosphorylation on Tyr-154	Breast cancer	(22)
ATM	Kinases	Phosphorylation on Ser-121 and Thr-127	DNA damage response	(35)
DAPK1	Kinases	Phosphorylation on Ser39	Acute Kidney Injury	(64)
UBC9	SUMO conjugating enzyme	Sumoylation on Lys-202, Lys-266, Lys-295, Lys-297, and Lys-303	Sumoylation	(65)

Several microRNAs (miRNAs) have been identified as post-transcriptional regulators of Peli1, targeting its 3’-untranslated region (3’ UTR) and repressing its expression (**Table 2**). MiRNAs are small, non-coding RNA molecules that regulate gene expression at the post-transcriptional level by binding to the 3’ UTR of target genes (67). MiRNAs are closely associated with various biological functions and pathological processes, including differentiation, metabolism, aging, autophagy, cell proliferation, and apoptosis (68).

### 3.2 Post- translational modifications of Peli1

Peli1 activity is modulated by various post-translational modifications, including phosphorylation, ubiquitination, and SUMOylation (**Table 2**). Peli1 can be interconverted between its inactive and active forms through a reversible phosphorylation mechanism, and various kinases can phosphorylate Peli1 and enhances its E3 ligase activity (63). IRAK1/IRAK4 and TBK1/Ikkϵ are the primary kinases responsible for Peli1 phosphorylation (7, 36). IRAK1 interacts with the FHA structural domain and phosphorylates the phosphorylation sites (Ser-76, Ser-78, Ser-80, Ser-82, and Thr-86) clustered within the “wing” of Peli1 (63). TBK1/IkappaB kinase (IKK) regulates the phosphorylation at Ser-76, Thr-288, and Thr-288 of Peli1 (36). The epidermal growth factor receptor (EGFR) leads to Peli1 phosphorylation at Tyr-154 (22). In the DNA damage response context, Ser-121 and Thr-127 phosphorylation by ataxia-telangiectasia mutated (ATM) kinase activates Peli1 ubiquitination activity (35). Death-associated protein kinase 1 (DAPK1) phosphorylates Peli1 at Tyr-154, destabilizing it and releasing the TRIF-RIP1 signalosome (64).

IRAK1 promotes auto- and mutual-polyubiquitination of Peli1 in a kinase-dependent manner and induces kinase-dependent Peli1 degradation. However, the precise mechanism governing the phosphorylation-mediated auto-Ub proteasome-dependent Peli1 degradation remains unclear (7).

Peli1 can undergo SUMOylation, a process in which the small Ub-related modifier (SUMO)- conjugating enzyme UBC9 binds and modifies Peli1 (65). Five lysine residues (Lys-202, Lys-266, Lys-295, Lys-297, and Lys-303) in Peli1 serve as SUMO-1 receptor sites, partially overlapping with lysine residues involved in ubiquitination (Lys-169, Lys-202, and Lys-266), suggesting competition between SUMOylation and ubiquitination (65).

## 4 Biological processes involving Peli1

### 4.1 Signaling pathways

**Figure 1:**
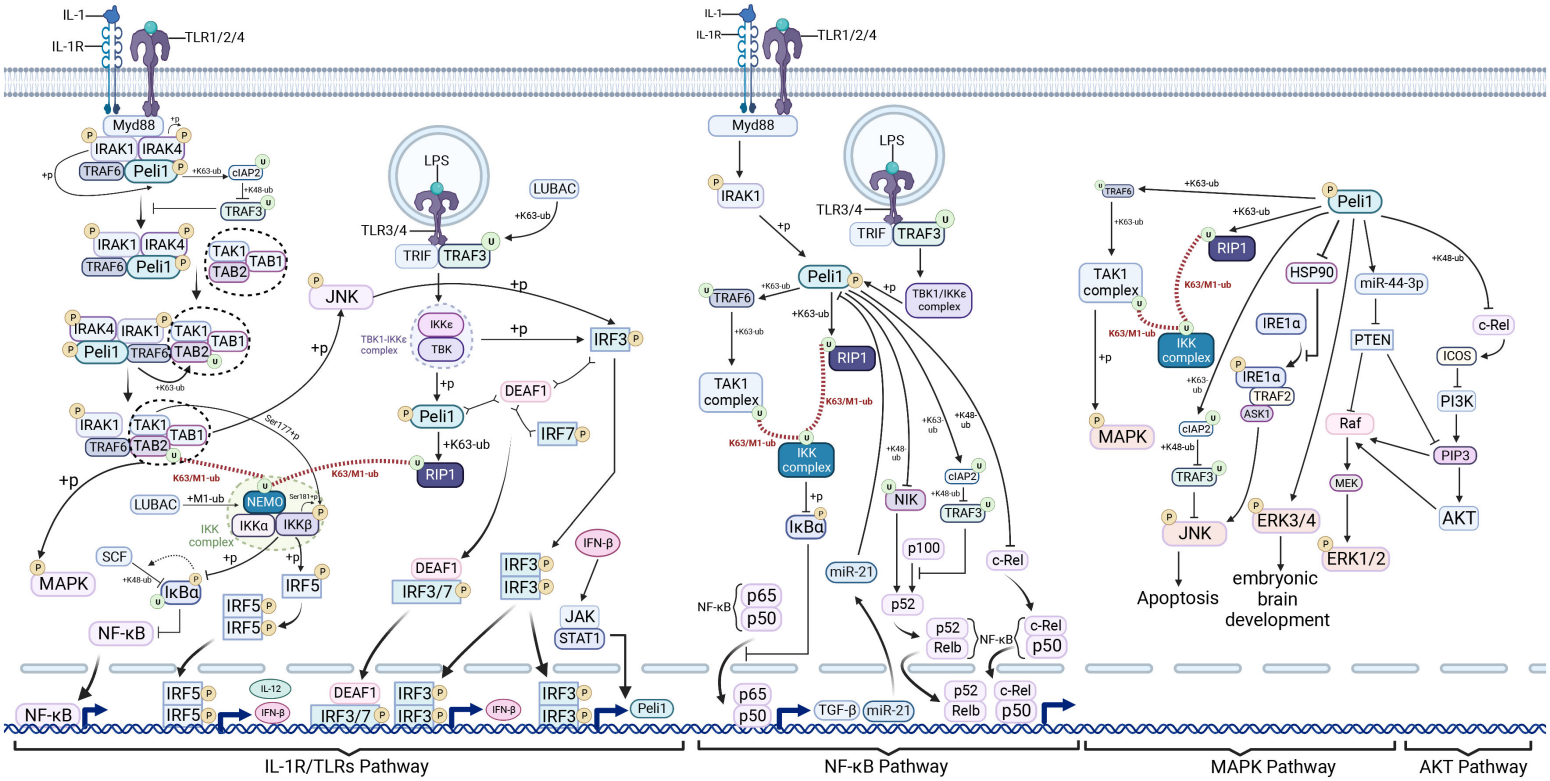
Pellino1 in interleukin-1 receptors/Toll-like receptors, nuclear factor−κB, mitogen-activated protein kinase, and AKT pathways. Pellino1 (Peli1), activated by interleukin-1 receptor-associated kinase (IRAK) phosphorylation, promotes the degradation of tumor necrosis factor receptor-associated factor 3 (TRAF3) by mediating cellular inhibitor of apoptosis protein 2 (cIAP2) ubiquitination in the myeloid differentiation primary response protein 88 (MyD88)-dependent interleukin-1 receptors (IL-1R)/Toll-like receptors (TLRs) signaling pathway. This process facilitates the cytoplasmic translocation of IRAK1-IRAK-TRAF6-PELI1 (an intermediate complex). The transforming growth factor-β (TGF-β)-activated kinase 1 (TAK1) complex forms complex II (IRAK1-IRAK-TRAF6-PELI1-TAK1-TAB1-TAB2) in conjunction with an intermediate complex that subsequently assembles into TRAF6-TAK1-TAB1-TAB2 (complex III). TRAF6 mediates TAK1 complex ubiquitination and recruits the IkappaB kinase (IKK) complex via the K63/M1-UB Ub chain, ultimately activating nuclear factor−κB (NF-κB) and mitogen-activated protein kinase (MAPK) signaling, and stimulating interferon regulatory factor 5 (IRF5) to induce interferon beta (IFN-β) production. In the Toll-interleukin-1 receptor-domain-containing adaptor-inducing IFN-beta (TRIF)-dependent Toll-like receptor (TLR)3/4 pathway, phosphorylated Peli1, in conjunction with the TBK1-Ikkϵ complex, mediates receptor-interacting protein kinase 1 (RIP1) ubiquitination, enabling RIP1 to recruit the IKK and TAK1 complexes through the K63/M1-UB Ub chain. This cascade activates NF-κB and MAPK signaling. The TBK1-Ikkϵ complex and Jun N-terminal kinase (JNK), activated by the TAK1 complex, can phosphorylate and activate IRF3, promoting IFN-β and Peli1 transcription. Peli1 enhances the positive feedback loop of IFN-β production. Peli1 promotes NF-κB canonical signaling by activating the IKK complex through TRAF6 and RIP1. NF-κB induces elevated miR-21 expression, inhibiting Peli1 expression, potentially forming a negative feedback loop. Peli1 mediates the ubiquitin-proteasome-dependent degradation of NIK, thus inhibiting NF-κB non-canonical signaling. However, Peli1 can also promote p100 to p52 processing and, ultimately, NF-κB non-canonical signaling by inhibiting TRAF3. Peli1 mediates ubiquitin-proteasome-dependent degradation of c-Rel. Peli1 activates the TAK1 complex via TRAF6 and RIP1, activating MAPK. Peli1 also inhibits TRAF3 via CIAP2, promoting JNK activation. Peli1 facilitates IRE1α phosphorylation by binding to HSP90, resulting in JNK activation. Peli1 inhibits phosphatase and tensin homolog (PTEN) by upregulating miR-44-3P, relieving Raf and phosphatidylinositol 3,4,5-trisphosphate (PIP3) repression, and ultimately activating extracellular signal-regulated kinase (ERK)1/2. In addition, Peli1 inhibits inducible co-stimulator (ICOS) through C-REL inhibition and activates phosphoinositide 3-kinase (PI3K), promoting PIP3 and AKT activation. Created using BioRender.com.

Peli1 plays a role in multiple signaling pathways upon stimulation by various factors, including IL-1R, TLRs, NF-κB, MAPK, and PI3K/AKT pathways, and participates in their crosstalk (**Figure 1**).

#### 4.1.1 IL-1R pathway

Peli1 plays a pro-inflammatory role in a variety of diseases through participation in the IL-1 pathway, where Peli1 was initially identified as interacting with IRAK homologous proteins (3). Peli1 participates in IL-1R signaling and to has limited modulation of IL-1 signaling (**Figure 1**). Upon IL-1 stimulation, the adaptor protein myeloid differentiation primary response protein 88 (MyD88) is recruited by the IL-1 receptor complex. Subsequently, the receptor complex recruits serine-threonine kinases IRAK4 and IRAK1 (69, 70). IRAK4 undergoes dimerization facilitated by MyD88, leading to IRAK4 trans-autophosphorylation (71). IRAK mediates TNF receptor-associated factor 6 (TRAF6) hyperphosphorylation, promoting its recruitment to the receptor complex (72). Peli1 interacts with IRAK4 and IRAK1 (72) and directly binds to TRAF6 and transforming growth factor-β (TGF-β)-activated kinase 1 (TAK1) (73). Ultimately, this binding results in MyD88-IRAK4-IRAK1-Peli1-TRAF6 signaling complex formation (45, 72, 74). Translocation of this complex from the membrane-bound receptor complex to the cytoplasm, necessary for MyD88-dependent signal transduction to TAK1 (75), involves Peli1-mediated TRAF3 degradation (44). Peli1 Phosphorylation by IRAK1 upon activation enhances its stability through K63-linked ubiquitination of the cellular inhibitor of apoptosis protein 2 (cIAP2). This event follows the cIAP2-mediated K48-linked Ub proteasome-dependent degradation of TRAF3, relieving cytoplasmic translocation inhibition of the MyD88-associated multiprotein complex by TRAF3 (44). Complex II (IRAK1-IRAK-TRAF6-Peli1-TAK1-TAB1-TAB2) forms the intermediate complex that transitions into complex III (TRAF6-TAK1-TAB1-TAB2) (72). The IKK complex, comprising IKKα, IKKβ, and the nuclear factor-κB essential regulator (NEMO), is a key component (76). TRAF6 mediates the ubiquitination of the K63-linked TAB2 subunit after M1-Ub chain formation from the NEMO subunit of the IKK complex catalyzed by the linear Ub chain assembly complex. The formation of K63/M1-Ub hybrids allows for the co-recruitment of both kinase complexes to the same Ub chain. TAK1 phosphorylates IKKβ at Ser-177, then autophosphorylated at Ser-181 of IKKβ, culminating in IKKβ activation (76). IKKβ has a dual role in the TLRs pathway: first, it recruits the E3 ligase SCF through IκBα phosphorylation, leading to the K48-linked IκBα ubiquitination and degradation through IκBα phosphorylation. This results in the derepression of P65 and P50, allowing their entry into the nucleus and activating of the NF-κB signaling pathway (76). Second, IKKβ phosphorylates the transcription factor interferon regulatory factor 5 (IRF5) at Ser-462, leading to its dimerization and nuclear translocation, thereby initiating the transcription of genes encoding major inflammatory cytokines such as IL-12 and IFN-β (76). TAK1 also induces MAPK phosphorylation (44). TAK1-mediated phosphorylation of Jun N-terminal kinase (JNK) activates IRF3 (77), suggesting that JNK activation facilitates crosstalk between MyD88-independent and dependent pathways associated with IRF3 activation (44).

TGF-β-BMP induces Smad6/Smad7, inhibiting the MyD88-IRAK4-IRAK1-Peli1-TRAF6 signaling complex by binding to different Peli1 regions. Consequently, the IL-1R-TLR signaling pathway is inhibited (45, 74). Moreover, the formation of the Smad6-A20 complex, involving Smad6 and the deubiquitinating enzyme A20, enhances the association of A20 with TRAF3 and TRAF6, thereby inhibiting IL-1 signaling (44).

However, Peli1 overexpression in human embryonic kidney cells (293 cells) did not affect cJNK and ELK1 expression (73). Peli1 deletion does not impact IL-1β-stimulated IKK activation, and Peli1 knockdown in primary epithelial cells does not alter the response to IL-1. Functional redundancy among Peli family proteins may account for these findings (78).

#### 4.1.2 TLRs pathway

The innate immune system encompasses multiple pattern recognition receptors responsible for sensing the pathogen-associated molecular patterns of invading pathogens, thus initiating an efficient innate immune response. Peli1 has been implicated in various TLR signaling pathways (**Figure 1**) (79). Several ligands of MyD88-dependent TLRs, including lipopolysaccharide (LPS) (for TLR4), CpG (for TLR9), R837 (for TLR7), and Pam3CSK4 (for TLR1 and TLR2), activate pathways consistent with IL-1R-dependent MyD88 (80). Peli1 also functions in the TLR3/4-dependent TRIF signaling pathway. TLRs dimerize in endosomes upon TLR3/4 stimulation by the corresponding ligands (e.g., TLR3: poly IC, viral double-stranded RNA; TLR4: LPS) (81) and recruits TRIF and TRAF3 (82, 83). H omologous to the E6-associated protein carboxyl terminus domain containing 3 mediates the K63-linked ubiquitination of TRAF3 at K138 (84), and TRAF3 ubiquitination or auto-ubiquitination (75) is necessary for binding and activation of the TBK1/Ikkϵ complex (83). Subsequently, the TBK1-Ikkϵ complex phosphorylates Peli1, activating its E3 ligase activity (27, 36, 85). Peli1 mediates TBK1 K63-Ub, resultING in a bidirectional signaling pathway that induces TBK1/Ikkϵ-mediated Peli1 phosphorylation. Activated Peli1 ubiquitinates RIP1 at K115 via K63 linkages (27, 28). Consequently, the TAK1 and IKK complexes are recruited to the polyubiquitin chains through K63-Ub of TABs and M1-Ub of NEMO, respectively (27). The proximity between TAK1 and IKKs facilitates TAK1-mediated IKKs phosphorylation and subsequent NF-κB activation (27). TAK1-mediated JNK phosphorylation activates IRF3 (44), suggesting that crosstalk between MyD88-independent and -dependent pathways associated with IRF3 activation occurs through JNK activation (77). Thus, Peli1 serves as a critical intermediary molecule in the TRIF-dependent NF-κB activation and the inducing pro-inflammatory genes (27). The TBK1-Ikkϵ complex regulates IFN-β and Peli1 transcription by inducing IRF3 phosphorylation. IFN-β can moderately increase Peli1 transcription through the JAK/STAT1 pathway (36). Furthermore, wild-type Peli1 might negatively regulate STAT1 expression, possibly preventing JAK-STAT1/2 pathway overactivation (86). This negative feedback regulation may be part of the complex regulatory mechanisms involving Peli1. However, further investigations are required to elucidate these mechanisms fully.

Inducing IFN-β is a substantial implications of the TRIF-dependent signaling pathway of TLRs. However, the mechanisms of IFN regulation by Peli1 are yielded complex and contradictory. Virus-stimulated secretion of IFN-β relies on a positive feedback loop, reduced IRF7, IFN4, and IFN6 mRNA production, and diminished IFN secretion in mice overexpressing Peli1 lacking ligase activity. T he positive feedback loop components we are also diminished, suggesting that Peli1 positively regulates IFN-β expression through a positive feedback loop (86). The synthetic product Smaducin-6 disrupts IKKe/TBK1/Peli1 and RIR1/Peli1 complexes by binding to Peli1, reducing IFN-β1 in immune cells (87). Therefore, Peli1 is necessary for interferon production in the viral double-stranded RNA reactions.

The interaction between Peli1 and IRF3, and their association with the IFN-β promoter, facilitates inducing IFN-β expression (36). This process is influenced by DEAF1, which binds to Peli1, and Peli1 phosphorylation can potentially impede this binding (43). DEAF1 is essential for the initial phase of TLR3-dependent IFN-β production after viral stimulation. DEAF1 enters the nucleus alone or forms a heterodimer with IRF3 or IRF7, binding to the IFN-β promoter to stimulate its transcription. The interaction of DEAF1 with the IFN-β promoter requires the presence of IRF3 (43). In contrast, Peli1 negatively regulate TLR-mediated IFN-I induction in microglia by inhibiting signaling events associated with TBK1 and Ikkϵ activation. Peli1 knockdown may enhance its antiviral capacity (88). *In vitro* restimulation of CD4+ and CD8+ splenic T cells in Peli1-/- mice with West Nile virus (WNV)-specific peptides increased IFN-γ production compared to wild-type mice. However, WNV infection in dendritic cells of Peli1-/- mice led to reduced IFN-β levels compared to wild-type mice (89). These findings suggest that Peli1 in different cells may be dominated by different regulatory pathways, exhibiting distinct IFN regulatory directions.

#### 4.1.3 NF-κB pathway

Peli1 influences the classical NF-κB pathway by engaging in the IL-1R/TLR pathway (**Figure 1**). Peli1 triggers TAK1 activation through TRAF6 within the MyD88-dependent pathway, facilitated by K63-Ub of TRAF6 (90–92). Peli1 facilitates RIP1 recruitment to the IKK complex and TAK1 in the TRIF-dependent pathway via the K63/M1-Ub hybrid chain (91, 92). The IKK complex induces IκBα phosphorylation, leading to its degradation via SCF-mediated ubiquitination, enabling the nuclear translocation of canonical NF-κB molecules, p50/p65 (76). Peli1 participates in a negative feedback loop of NF-κB during liver regeneration, wherein NF-κB upregulates miR-21 precursor transcripts that target Peli1, suppressing the NF-κB pathway (51). Peli1 negatively regulates NF-κB in T cells by mediating the Ub proteasome-dependent degradation of the late phase NF-κB protein, c-Rel (13, 58, 93), preventing excessive NF-κB activation (13, 52).

In the non-canonical NF-κB pathway, Peli1 displays contrasting regulatory functions depending on the context. Peli1 acts as a negative regulator of esophageal squamous carcinoma’s radiotherapy sensitivity and lupus erythematosus syndrome by promoting the Ub proteasome-dependent NIK degradation, inhibiting the nuclear translocation of non-canonical NF-κB effector molecules, p52, and Rel B (15, 16). Peli1 also augments cIAP2 stability by facilitating the K63-linked cIAP2 ubiquitination (25, 94, 95), enabling cIAP2 to mediate the K48-Ub and TRAF3 degradation (26), thereby alleviating the inhibition of p100 processing into p52 by TRAF3 (59). Consequently, Peli1 promotes p52 entry into the nucleus and relieves the inhibition of the classical NF-κB molecule p65 owing to p100 accumulation (59).

#### 4.1.4 MAPK pathway

Peli1 influences the MAPK pathway through multiple mechanisms (**Figure 1**), regulating inflammation and apoptosis and participating in brain development and angiogenesis (61, 85, 94, 96, 97). Peli1 facilitates TRAF6 and RIP1 ubiquitination in the IL-1R/TLR pathway (29, 98), leading to P38 activation via TAK1 (99). Moreover, Peli1 mediates the K63-Ub of cIAP2, activating JNK and P38 by via increasing cIAP2-mediated TRAFT3 degradation (26, 80). Peli1 induces miR-494-3p expression in cardiomyocyte exosomes, inhibiting its target, phosphatase and tensin homolog (PTEN), and activating the AKT, Smad2/3, and extracellular signal-regulated kinase (ERK) signaling pathways (100). Peli1 activates the MAPK pathway by modulating endoplasmic reticulum (ER) stress. By binding to heat shock protein 90 (HSP90), Peli1 hinders the interaction of HSP90 with IRE1α, a key protein in ER stress, promoting phosphorylation-dependent IRE1α activation (47). Activated IRE1α facilitates TRAF2 recruitment, further promoting p-JNK activation through apoptosis signal-regulating kinase 1 inducing downstream apoptotic signaling (47, 101). IRE1α promotes x-box binding protein 1 (XBP1) splicing and maturation (47), while p38MAPK phosphorylates XBP1 at Thr-48 and Ser-61, augmenting the induction of apoptotic signaling and nuclear migration of XBP1 in mice (102).

#### 4.1.5 AKT pathway

Peli1 activates the AKT pathway and is involved in various biological and pathological processes, including cancer progression (23, 103), drug resistance (104), angiogenesis (96, 100, 105), T follicular helper (Tfh) cell differentiation (93), microglia activation (98), glycolysis, and macrophage M1 polarization (31). Peli1 regulates AKT activationvia several pathways: 1) Peli1 inhibits the inducible co-stimulator (ICOS) and suppresses the PI3K/AKT pathway downstream of ICOS by mediating ubiquitination-dependent c-Rel degradation (93); 2) Peli1 upregulates miR-494-3p expression in cardiomyocyte exosomes and inhibits its target PTEN. This inhibition prevents PTEN-mediated phosphatidylinositol 3,4,5-trisphosphate (PIP3) dephosphorylation, activating downstream AKT/endothelial nitric oxide synthase/nitric oxide signaling (100).

### 4.2 Cell death

**Figure 2:**
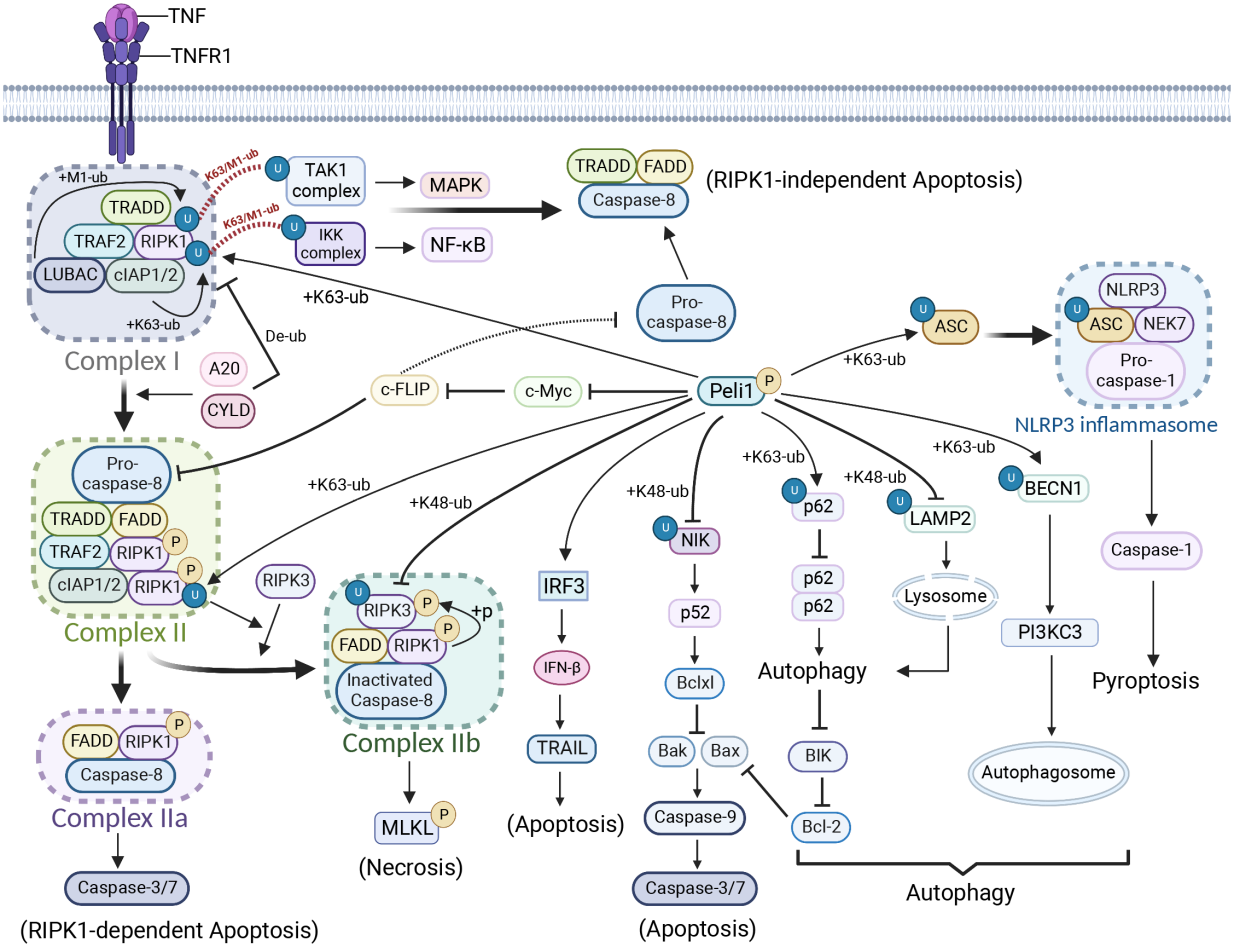
Peli1 regulation in cell death and autophagy. Upon tumor necrosis factor (TNF) stimulation, the TNFR1-associated death structural domain (TRADD) recruits cIAP1 and cIAP2 to RIPK1 via the TRAF2 and linear ubiquitin chain assembly complexes, forming complex I. Rapid ubiquitination of RIPK1 occurs. Complex I is deubiquitinated by A20 and cylindromatosis, forming complex II. Complex II bifurcates into complex IIa, triggering RIPK1-dependent apoptosis, and complex IIb, which induces necrosis. Complex I forms a TRADD-FADD-caspase-8 complex, leading to RIPK1-independent apoptosis. PELI1 preferentially recruits RIPK3 to form complex IIb by mediating RIPK1 ubiquitination in complex II. However, PELI1 mediates the ubiquitin-proteasome-dependent degradation of RIPK3, thereby inhibiting necrosis. PELI1 inhibits RIPK1-dependent or RIPK1-independent apoptosis by suppressing c-myc and promoting c-FLIP (a catalytically inactive caspase-8 homolog) expression. However, Peli1 can also promote apoptosis through tumor necrosis factor-related apoptosis-inducing ligand by upregulating IFN-β expression. Peli1 inhibits NF-κB non-classical signaling and downstream B-cell lymphoma-extra large via NIK, promoting apoptosis. Peli1 inhibits autophagy by mediating p62 ubiquitination, resulting in BIK accumulation, which activates downstream apoptotic signals. Peli1 inhibits lysosome formation and autophagic signaling by mediating the ubiquitin-proteasome-dependent degradation of lysosome-associated membrane protein 2. However, Peli1 can also promote autophagosome production by mediating the ubiquitination of K63-linked Beclin 1. In addition, Peli1 induces NLRP3 inflammasome production and pyroptosis through the ubiquitin-mediated modification of apoptosis-associated speck-like protein (ASC). Created using BioRender.com.

Peli1 regulates cell death through various pathways, including apoptosis, necroptosis, and pyroptosis (**Figure 2**). Peli1 plays a dual regulatory role in necrosis and apoptosis by modulating the ubiquitination of RIPK1 (RIP1) and mRNA levels of cellular FLICE-like inhibitory protein (c-FLIP) (28). Upon TNF stimulation of tumor necrosis factor receptor 1 (TNFR1), RIPK1 is recruited to the TNFR1-associated death structural domain (TRADD), which recruits cIAP1 and cIAP2 via the TRAF2 and linear ubiquitin chain assembly complexes (LUBAC), culminating in complex I formation (28, 106). In complex I, RIPK1 undergoes rapid Ub chain polymerization, activating the TAK1 and IKK complexes through the K63/M1-Ub hybrids, inducing NF-κB/MAPK pathway activation. K63 and M1 ubiquitination of RIPK1 contributes to recruiting Peli1 to complex I. Without an A20-binding inhibitor of NF-κB1 (ABIN1), Peli1 further promotes K63-linked RIPK1 ubiquitination (28, 106). A20 and cylindromatosis (CYLD) mediate complex I degradation by deubiquitinating its components to form complex IIa or IIb. Complex IIa comprising the Fas-associated death domain (FADD), caspase-8, and RIPK1, triggers RIPK1-dependent apoptosis by activating caspase-8, -3, and -7 (106). TRADD in complex I recruits FADD and caspase-8 to initiate RIPK1-independent apoptosis. C-FLIP, a catalytically inactive caspase-8 homolog, interacts with procaspase-8 and impedes caspase-8 processing by counteracting the cytotoxic activity of complex IIa. Peli1 upregulates c-FLIP mRNA levels by inhibiting the repressive transcription factor c-Myc to inhibit RIPK1-dependent and RIPK1-independent apoptosis (28, 107). Upon caspase-8 inactivation, RIPK1 binds to the RIP homotypic interaction motif (RHIM) of RIPK3 to form complex IIb. This complex promotes RIPK3 oligomerization and phosphorylation, which recruits and phosphorylates Mixed lineage kinase domain-like protein (MLKL), resulting in membrane perforation and programmed necrosis (28, 106). Peli1 mediates the K63-linked ubiquitination of RIPK1 in complex II, and deleting Peli1 inhibits RIPK3, RIPK1, and FADD binding, and RIPK3 and MLKL phosphorylation, indicating the indispensable role of Peli1 in the RIPK1-RIPK3 necrosome (28). However, how Peli1 exerts its ubiquitination function requires further investigation.

Under hypoxic LPS conditions, DAPK1 phosphorylates Ser-39 of Peli1, destabilizes it, and reduces the binding between Peli1 and RIPK1. The release of the TRIF-RIPK1 signalosome enhances RIPK1 binding to caspase-8, ultimately inducing apoptosis in renal tubular cells (64). Surprisingly, T182 phosphorylation on RIPK3 preferentially recruits Peli1, leading to the K48-linked ubiquitination-dependent degradation of kinase-active RIPK3 (12, 108), possibly functioning as a feedback mechanism. Although much progress has been made, further investigations are needed to uncover additional mechanisms by which Peli1 is involved in RIPK1-mediated necrosis and apoptosis.

In contrast, Peli1 promotes apoptosis through alternative pathways (61, 94, 109–111). Peli1 inhibits ionizing radiation-induced (IR) activation of the non-classical NF-κB pathway by promoting NIK degradation, thereby preventing IR-induced expression of B-cell lymphoma-extra large. Consequently, caspase-9 maturation and apoptotic signaling are restored (16). Peli1 mediates K63-linked ubiquitination of the autophagy-associated protein P62 at residue K7, disrupting P62 homodimer formation and autophagic degradation. Furthermore, Peli1 upregulates BIK expression by inhibiting BIK autophagic degradation, inhibiting the apoptosis suppressor protein B-cell lymphoma 2 (BCL2) and ultimately promoting apoptosis (41). Peli1 upregulates IFN-β expression by promoting IRF3/IRF5, and IFN-β1 augments the tumor necrosis factor-related apoptosis-inducing ligand in human peripheral blood T cells, inducing apoptosis in septic cells (87).

The inflammatory vesicle complex NACHT, LRR, and PYD domains-containing protein 3 (NLRP3) comprises the ligand-sensing receptor NLRP3, adapter protein apoptosis-associated speck-like protein (ASC), pro-caspase-1, and the regulatory protein NIMA-associated kinase 7. Peli1 facilitates K63-Ub at K55 of ASC at the inflammasome junction, promoting ASC/NLRP3 interactions and ASC oligomerization for inflammasome activation and pyroptosis induction (40).

### 4.3 Autophagy

Peli1 demonstrates dual regulation of autophagy depending on specific circumstances. T *in vivo in vitro* the E3 ligase activity of Peli1 was significantly increased under *in vivo* and *in vitro* reoxygenation conditions (**Figure 2**). On the one hand, Peli1 promotes cardiomyocyte death by impairing P62 autophagic degradation and reducing autophagic flux (41). On the other hand, inhibiting BIK autophagic degradation promotes the activation of the apoptotic pathway by inhibiting BCL2 (41). T he human immunodeficiency virus (HIV) protein Tat induces increased Peli1 expression, mediating the K63-Ub of Beclin 1, leading to increased autophagy and disruption of the blood-brain barrier (BBB) through reduced tight junctional zonula occludens-1 (ZO1). In Parkinson’s disease, Peli1 is upregulated in microglia by extracellular preformed fibrils (PFF) of α-synuclein, resulting in reduced lysosomes and blocked autophagic flux due to Ub proteasome-dependent lysosome-associated membrane proteins degradation (18). Overall, Peli1 regulates autophagy through diverse pathways, necessitating further investigations to determine the underlying regulatory mechanisms depending on the specific context.

### 4.4 DNA damage repair

**Figure 3:**
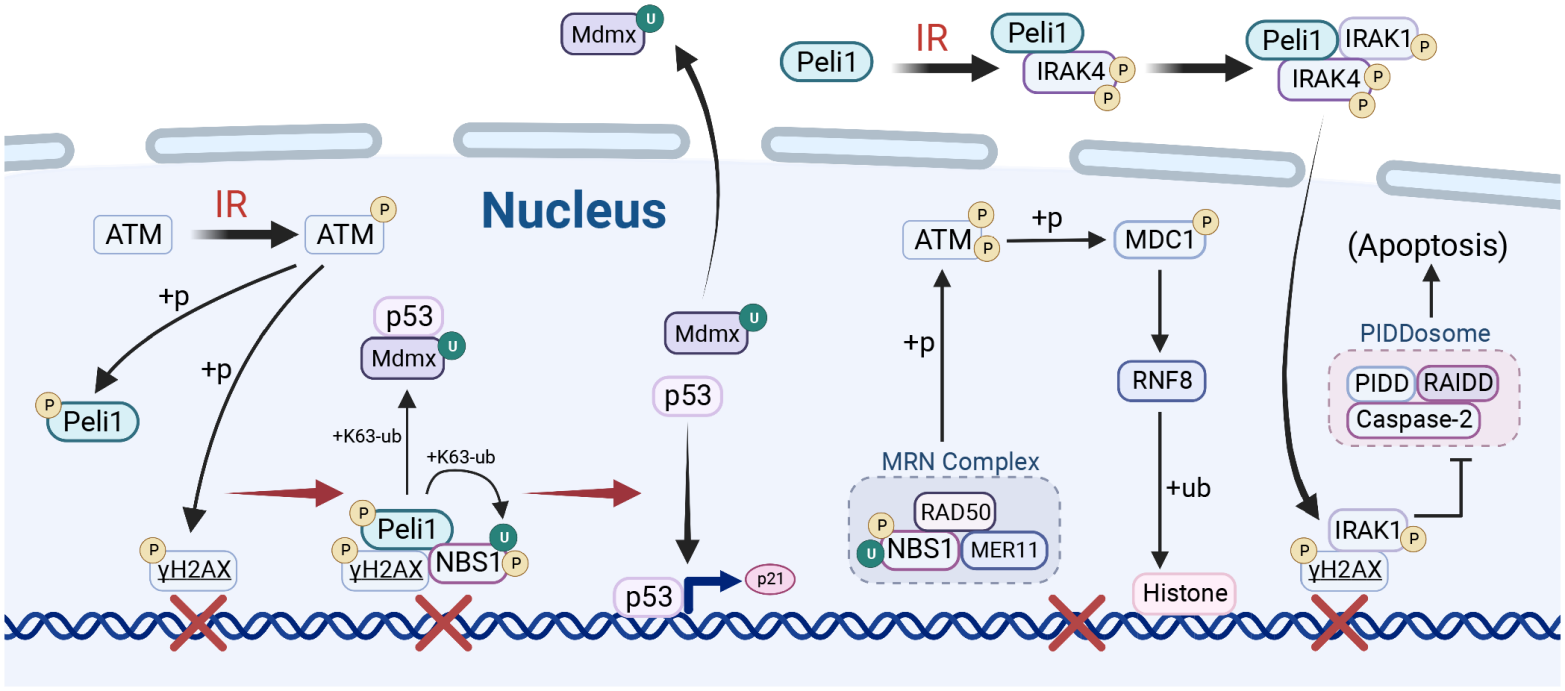
Peli1 in DNA damage repair. Upon ionizing radiation (IR) stimulation, PELI1 mediates nibrin (NBS1) ubiquitination and facilitates MRE11-RAD50-NBS1 (MRN) complex assembly, which is recruited to the DNA double-strand break (DSB) site by γH2AX and activated by ataxia-telangiectasia mutated (ATM) phosphorylation. The MRN complex enhances ATM phosphorylation, which mediates the phosphorylation of MDC1, thereby promoting the ubiquitination of histones by RNF8/RNF18 and establishing a platform for DNA damage repair. Peli1, recruited to the DSB site by γH2AX, promotes the nuclear export of murine double minute X (MDMX) by mediating MDMX ubiquitination, thus liberating P53 to activate the transcription of downstream genes in response to DNA damage repair. Peli1 binds to IRAK4 to promote IRAK1 recruitment and phosphorylation, which then translocates to the nucleus and co-localizes with γH2A, inhibiting the pro-apoptotic PIDDosome complex. Created using BioRender.com.

DNA double-strand breaks (DSBs) signaling and repair are crucial for maintaining genomic integrity. Peli1 is crucial in DNA damage-responsive protein accumulation and efficient homologous recombination (HR) repair, making it essential for DSB-responsive pathways (**Figure 3**) (33–35, 46). Peli1 participates in multiple pathways associated with the DNA damage response (35). Peli1 is recruited to DSB sites by γH2AX and activated through ATM phosphorylation.DSBs activate ATM upon laser micro-irradiation-induced DNA damage, which phosphorylates H2A histone family member X (H2AX) to its activated state, γH2AX. Peli1 binds to nibrin (NBS1), also recruited to DSBs by γH2AX. Peli1 mediates the K63-Ub of NBS1 at the K686 and K690 sites, leading to MRE11 and RAD50 recruitment to form the MRE11-RAD50-NBS1 (MRN) complex. The MRN complex phosphorylates and activates ATM, activating MDC1 and its partner RNF8 recruited by γH2AX. This activation leads to RNF8/RNF168 recruitment and establishing of a platform for downstream DNA repair proteins by mediating histone ubiquitination (112). Additionally, Peli1, recruited by γH2AX to the site of DNA damage, binds to phosphorylated P53 at the Thr-18 site through its FHA domain, facilitating the ubiquitination of the P53 repressor protein MDMX via K63 linkage. U biquitination promotes the nuclear export of MDMX and releases P53, which activates the transcription of downstream genes involved in DNA damage repair (DDR), such as P21 (33, 34). Peli1 binds to IRAK4 after IR and autophosphorylates Thr-345/Ser-346, independent of its E3 Ub ligase activity. This event recruits IRAK1 to Peli1 via IRAK4, leading to IRAK1 activation and nuclear translocation through Thr209 phosphorylation. Nuclear IRAK1 co-localizes with γH2AX and inhibits the pro-apoptotic PIDDosome complex (PIDD1-RAIDD-caspase-2), exerting an anti-apoptotic function (46).

### 4.5 Glycolysis

Peli1 exerts a complex and context-dependent regulation of glycolysis through various mechanisms. Peli1 is reportedly a negative regulator of glycolysis (37, 39, 52); however, other studies have shown that Peli1 promotes glycolysis (31). Peli1 enhances the stability of the tuberous sclerosis 1 (TSC1)/TSC2 complex in tumor-infiltrating CD8+ T cells by mediating K63-linked TSC1 ubiquitination (39). Peli1 inhibits TSC2 phosphorylation and inactivation by TCR/CD28 via the AKT pathway. Consequently, the stabilized complex hampers mammalian target of rapamycin complex 1 (mTORC1) activation by inactivating Rheb. Peli1 suppresses the phosphorylation of mTORC1 target proteins S6K and S6, accompanied by downregulating the downstream glycolytic genes, such as *GLUT1, HK2, PGK1, Eno1, Pkm, Hif1a*, and *Myc*. In pathogenic Th17 cells, Peli1 downregulates c-Myc by mediating the K48-Ub and ubiquitination-dependent degradation of c-Rel, a potent activator of genes involved in glycolysis and mitochondrial respiratory pathways (113), thereby inhibiting glycolysis (52). However, contradictory findings have been reported regarding glycolysis regulation by Peli1 in macrophages.

Peli1 promotes K63-linked ubiquitination of IRF5 in response to LPS/IFN-γ stimulation, enhancing glycolysis and M1 polarization by increasing the nuclear translocation and transcription factor activity of IRF5 (31). Conversely, Peli1-mediated K63-linked ubiquitination of IRAK1 and STAT1 activation inhibits IL-10-induced polarization of M2c macrophages and IL-10 production, thereby inhibiting tumor growth. Peli1-deficient bone marrow-derived macrophages exhibit defective mitochondrial respiration but enhanced glycolysis during M2c polarization (37). However, IL-10 inhibits LPS-induced glucose uptake and macrophage glycolysis (114). Therefore, the regulation of glycolysis by Peli1 in the tumor microenvironment and different cell types may involve additional pathways that warrant further in-depth investigation.

## 5 Diseases involved in Peli1

### 5.1 Tumors

**Figure 4:**
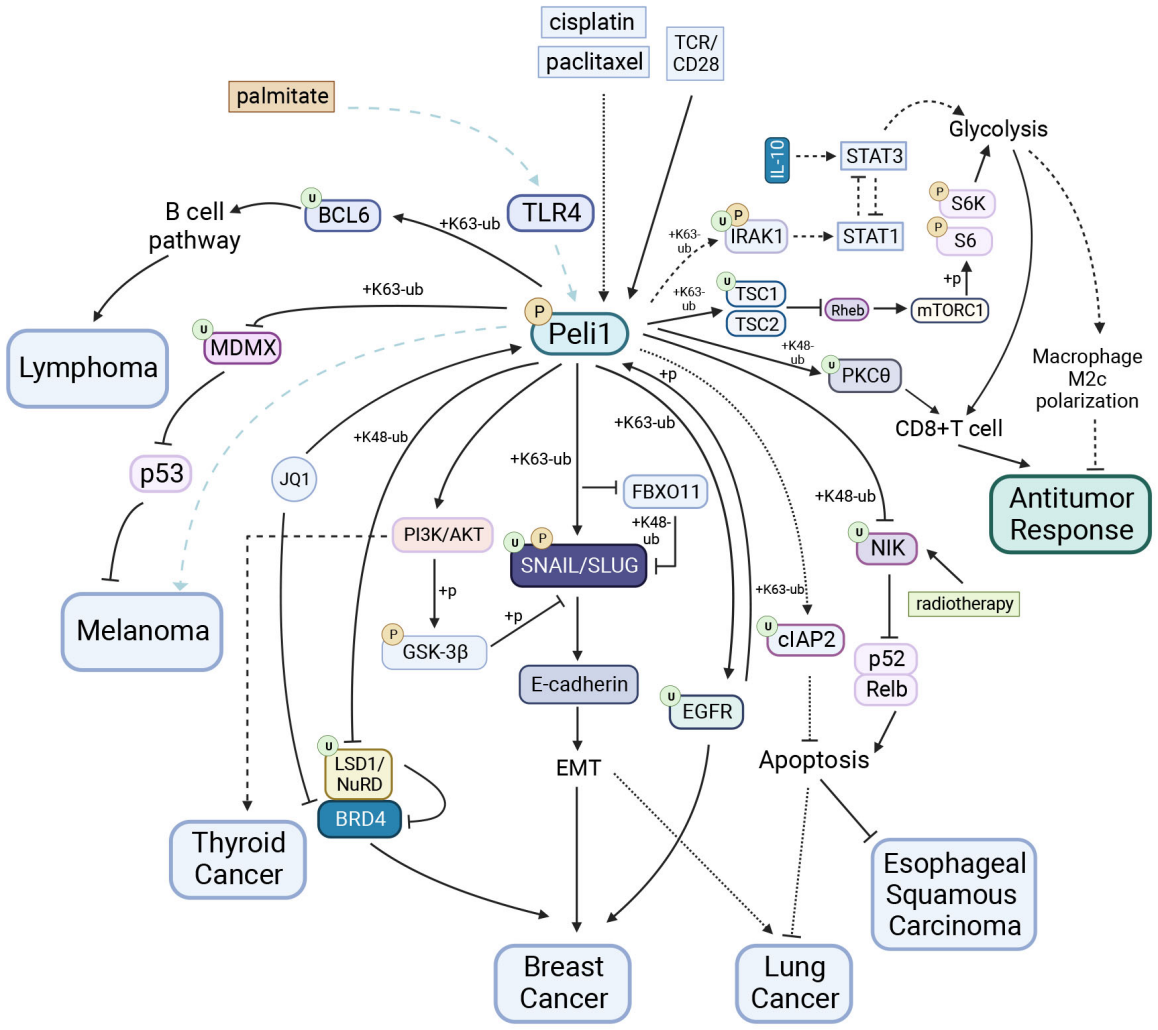
Peli1 in Tumors. Peli1 stabilizes SNAIL/SLUG via ubiquitination and the PI3K/AKT/GSK-3βpathway, contributing to breast and lung cancers EMT. Peli1 is detrimental to JQ1 drug tolerance in breast cancer through LSD1 degradation and the BRD4/LSD1/NuRD complex dissociation. Peli1 interacts with EGFR to promote breast cancer metastasis. Peli1 overexpression upregulates the expression of the apoptosis-inhibitory protein cIAP2, conferring resistance to cisplatin- and paclitaxel-induced apoptosis in lung cancers. Peli1 expression is upregulated in papillary thyroid carcinoma and promotes cancer cell proliferation and migration by activating the PI3K/AKT pathway. Elevated Peli1 expression induces lymphoma development by facilitating BCL6 ubiquitination and promoting the constitutive activation of the post-BCL6 B-cell signaling pathway. Peli1 is involved in palmitate-induced, TLR4-dependent lung metastasis in melanoma, whereas Peli1 inhibits melanoma progression through MDMX ubiquitination and cytoplasmic localization. Peli1 enhances the sensitivity of esophageal squamous carcinoma to radiotherapy by inhibiting the IR-induced activation of the non-canonical NF-κB pathway. Peli1 mediates PKCθubiquitination and inhibits glycolysis via TSC1 ubiquitination, negatively regulating antitumor activity in CD8^+^ T cells. In contrast, Peli1 exhibits positive antitumor capacity in macrophages by inhibiting glycolysis through IRAK1 TSC1 ubiquitination.Created using BioRender.com.

Peli1 plays a pivotal role in tumors, with complex roles across different cancer types, impacting tumor behavior, therapeutic response, and immune modulation (**Figure 4**). Understanding Peli1 functions holds promise for developing targeted cancer therapies and improving clinical management.

#### 5.1.1 Breast cancer

Triple-negative breast cancer (TNBC), which is aggressive and heterogeneous, is the second most common malignancy among women. Peli1 is upregulated in breast cancer tumor tissues, and higher Peli1 expression levels correlate with reduced survival rates. Peli1 is a positive regulator of tumor metastasis that significantly contributes to breast cancer mortality (24). Silencing Peli1 reduces tumor migration, invasion, and tumor-sphere formation. Loss of E-cadherin expression, a characteristic feature of epithelial-mesenchymal transition (EMT), is associated with the upregulation of E-cadherin transcriptional repressors (e.g., SNAIL, SLUG, TWIST, ZEB1, and ZEB2) (115–119). Peli1 stabilizes SNAIL/SLUG by mediating K63-linked polyubiquitination while inhibiting FBXO11 binding to SNAIL/SLUG and subsequent K48-linked Ub proteasome-dependent degradation (24). Peli1 inhibits glycogen synthase kinase-3 beta activity by activating AKT-mediated phosphorylation, disrupting the SCF-mediated ubiquitination degradation of SNAIL through SNAIL phosphorylation (105, 120). Peli1 interacts with EGFR to promote breast cancer metastasis. Activated EGFR phosphorylates and activates Peli1, enabling it to mediate the K63-linked EGFR ubiquitination and protect it from degradation. Understanding this process provides insights for refining therapies targeting EGFR (22).

Prognostic markers and biological therapies of Peli1 hold promise for improved disease management due to resistance to conventional therapies. Peli1 is detrimental to JQ1 drug tolerance by targeting bromodomain-containing protein 4 (BRD4). BRD4 is known for its role in the super-enhancer organization and the transcriptional activation of major oncogenes, including c-MYC and BCL2 (121, 122). BRD4 inhibitors, such as JQ1, have emerged as promising therapeutic agents for cancer (123). BRD4 unexpectedly interacts with the LSD1/NuRD complex and co-domains with this inhibitory complex at the super-enhancer, similar to the BRD4/LSD1/NuRD complex. However, long-term treatment with JQ1 leads to elevated Peli1 expression, resulting in the dissociation of the BRD4/LSD1/NuRD complex, LSD1 degradation via Ub-proteasome mechanisms, and JQ1 resistance development (21). Peli1 is associated with resistance to combination chemotherapy in breast cancer (21). Therefore, Peli1 plays a pathological role in breast cancer and contributes to chemotherapy tolerance, underscoring the importance of comprehensive investigations to enhance the clinical management of breast cancer.

#### 5.1.2 Lymphoma

Peli1 overexpression, implicated in lymphoma progression, is significantly elevated in various lymphomas. Peli1 expression may be a valuable prognostic indicator for patients with lymphomas, especially those with diffuse large B cell lymphoma (DLBCL). High Peli1 expression in lymphoma is associated with frequent bone marrow involvement and shorter relapse-free survival (124). Peli1 expression may be an independent prognostic indicator of DLBCL (30). Elevated Peli1 expression induces lymphoma development by facilitating B-cell lymphoma 6 (BCL6) ubiquitination through K63 linkage and promoting the constitutive activation of the post-BCL6 B-cell signaling pathway. This activation increases mature B cells and B220^+^ lymphocytes infiltration into the tumor (30). Peli1 expression positively correlates with the MYC, BCL6, BCL2, and MUM1 expression in lymphomas (30, 124). A study focusing on the Bcl-6-dependent risk stratification of DLBCL based on Peli1 nuclear expression highlighted the potential role of Peli1 and Bcl-6 in assessing DLBCL risk (125) Peli1 expression is highly elevated in high-grade lymphomas but significantly reduced in low-grade lymphomas (124). A completely different role for Peli1 in cHL exists, where miR-21-5p acts as an abundantly expressed oncogene that protects cHL from apoptosis by targeting Peli1 (109). This paradoxical role of Peli1 in different lymphomas may partly explain the differential expression of Peli1 in distinct grades of lymphomas and provide insights into developing t of personalized medical treatments for lymphomas.

#### 5.1.3 Lung cancer

Elevated Peli1 expression in lung cancer plays a crucial role in the development and progression and drug resistance (23, 25, 126). Peli1 expression is an essential prognostic indicator of survival in patient with lung cancer (126). First, Peli1 enhances the stability of SNAIL/SLUG by mediating SNAIL/SLUG polyubiquitination through K63 linkage, consequently inhibiting E-cadherin expression. This process promotes various malignant characteristics of lung cancer cells, including proliferation, survival, colony formation, invasion, and migration, by inducing EMT (23). Second, Peli1 overexpression upregulates the expression of the apoptosis-inhibitory proteins cIAP1 and cIAP2, conferring resistance to cisplatin- and paclitaxel-induced apoptosis in tumor cells. Peli1 achieves this effect by directly interacting with cIAP2 and stabilizing it through its E3 ligase activity, involving K63-linked polyubiquitination (25). The combination of low Peli1 expression and high necrosis factor RIPK3 expression along with DDR factor p53 is a significant predictor of survival in patients with stage I non-small cell lung cancer squamous cell carcinoma subtype (126). Therefore, Peli1 plays a pathological role in lung cancer and represents a novel therapeutic target to treat for this disease.

#### 5.1.4 Other tumors

The role of Peli1 varies across cancer types. Peli1 promotes tumor progression and sensitivity to radiotherapy in papillary thyroid and esophageal squamous carcinomas (16, 103). Conversely, its functions are contradictory in melanoma, suggesting a complex role in tumor biology (34, 127).

Peli1 expression is increased in papillary thyroid carcinoma and promotes cancer cell proliferation and migration by activating the PI3K/AKT pathway, possibly associated with the deletion of miR-30c-5P, which targets Peli1 (103).

However, Peli1 plays contradictory roles in melanoma progression. Peli1 mediates MDMX ubiquitination by binding to its RING structural domain, leading to MDMX cytoplasmic localization. This activation promotes P53-mediated tumor progression inhibition, with higher Peli1 expression associated with improved survival patients with melanoma (34). In contrast Peli1 is involved in palmitate-induced, TLR4-dependent lung metastasis in melanoma (127). The diverse roles of Peli1 in the different phases of melanoma progression warrant further investigation.

Peli1 also plays a protective role in esophageal squamous carcinoma; Peli1 enhances the sensitivity of esophageal squamous carcinoma to radiotherapy by inhibiting the IR-induced activation of the non-canonical NF-κB pathway. This effect is achieved through the Peli1-mediated NIK ubiquitination and degradation, leading to increased tumor cell apoptosis (16). Consistent with this, a comprehensive analysis of transcriptome and methylation data from the Gene Expression Omnibus database revealed that high Peli1 expression in in patients with esophageal cancer is associated with longer overall survival (128).

Furthermore, Peli1 exhibits opposing effects on different immune cells during antitumor responses. In CD8^+^ T cells, Peli1 negatively regulates antitumor activity. First, Peli1 mediates the ubiquitination degradation of PKCθ via K48 linkage (20), inhibiting T cell receptor signaling and impairing CD8^+^ T cell function. Second, Peli1 increases the stability of the TSC1/TSC2 complex by mediating the K63-Ub of TSC1 (39). This stabilization inhibited TSC2 phosphorylation and inactivation by TCR/CD28 signaling through the AKT pathway. Consequently, the stable complex suppressed mTORC1 activation and phosphorylation S6K and S6 by inactivating Rheb. Ultimately, these mechanisms lead to a reduced antitumor response in T cells. In contrast, Peli1 exhibits positive antitumor capacity in macrophages by inhibiting glycolysis. This is achieved by mediating the K63-linked IRAK1 ubiquitination, leading to STAT1 activation and the inhibition of M2c macrophage polarization induced by IL-10 (37). As a result, tumor growth was inhibited *in vivo*. Furthermore, Peli1 inactivates the mitotic spindle checkpoints by mediating K48-Ub and Ub degradation of BubR1, inducing extensive chromosomal aneuploidy and tumorigenesis (14).

### 5.2 Cardiovascular disorders

Peli1 is mainly pathogenic in infarction, primarily through mechanisms involving inflammation, autophagy, and apoptosis. Targeting Peli1 could rescue infarction and improve cardiac function (95–97, 105).

Inflammation is a crucial pathway through which Peli1 is involved in infarction. Peli1 silencing reduces inflammatory infiltration and ultimately improves cardiac dysfunction by regulating the TLR/IL1R pathway (92), inhibiting RIP1 and TRAF6 ubiquitination, and inhibiting NF-κB activity. Peli1 promotes the nuclear translocation of IRF5 by mediating the K63-Ub of IRF5, promoting M1 polarization of macrophages andcardiomyocyte death (32). Peli1 also regulates infarction via autophagy and apoptosis. Reoxygenation, *in vivo* and *in vitro*, significantly increases the E3 ligase activity of Peli1 (41). Furthermore, Peli1 promotes the ubiquitination of the autophagic protein P62 at residue K7 through K63 linkage. Disrupting P62 homodimer formation due to ubiquitination prevents P62 autophagic degradation, consequently reducing autophagic flux and promoting cardiomyocyte death. Inhibiting BIK autophagic degradation by Peli1 promotes apoptotic pathway activation by inhibiting BCL2, which increases myocardial infarction and contributes to cardiac dysfunction.

In an LPS-induced myocarditis model, Peli1 was activated and promoted pro-inflammatory genes expression (129). Si-Peli1 Treatment alleviated or even reversed LPS-induced cellular injury by modulating cardiomyocyte energy metabolism and altering the expression of key genes (*Cs, Cpt2*, and *Acadm*) and metabolites (3-oxoocotanoyl-CoA, hydroxypyruvic acid, lauroyl-CoA, and nicotinamide adenine dinucleotide phosphate) (129). In the context of diabetes-induced cardiovascular response, Peli1 elevates its interaction with HSP90, competitively inhibiting IRE1α binding to HSP90 and promoting IRE1α phosphorylation and ER stress. Peli1 also enhances TRAF2 recruitment to IRE1α by promoting XBP1 splicing and maturation, activating the MAPK pathway and mediating apoptosis of cardiac microvascular endothelial cells (47).

Peli1 has been proposed as a potential predictive biomarker for coronary artery disease (130). Peli1 plays a protective role in atherosclerosis as its deficiency increases pathogenic immune cell subsets (Th1 cells and Tfh cells)and decreases protective subsets (Treg and Breg cells), promoting systemic inflammation, immune cell infiltration, and foam cell formation in vascular smooth muscle cells, thereby exacerbating atherosclerosis (131). Peli1 is also downregulated in giant cell arteritis (132).

In summary, Peli1 is involved in cardiovascular disease progression by regulating inflammation autophagy, and apoptosis, thus providing novel insights and potential therapeutic targets for individualized treatment.

### 5.3 Infectious diseases

Peli1 plays distinct pathogenic and protective roles in response to various viral infections. Peli1 exerts pathogenic effects on central nervous system (CNS) antiviral infections (38, 88, 89). Peli1 negatively regulates TLR-mediated IFN-I induction by inhibiting TBK1/Ikkϵ activation-related signaling events. Peli1-deficient mice and microglia infected with vesicular stomatitis virus (VSV) exhibited significantly enhanced IFN-α/IFN-β expression and increased antiviral responses (88). Furthermore, Peli1 acts as a pathogenic factor during WNV infection by promoting cell attachment, entry, replication, and neuroinflammation via microglial activation (89). Peli1 is also involved in HIV invasion of the BBB, where Tat induces elevated Peli1 expression. Peli1 mediates the K63-linked ubiquitination of beclin 1, resulting in increased autophagy and disruption of the BBB through the disassembly of tightly linked ZO1 (38). The Japanese encephalitis virus (JEV) suppresses Peli1 in microglia by upregulating microRNA-155, increasing TRAF3 expression. This mechanism facilitates immune escape from JEV by inhibiting the non- canonical NF-κB pathway through NF-κBp100 accumulation (59). Peli1 promotes Zika virus (ZIKV) infection and placental inflammation and is involved in multiple stages of ZIKV infection, including cellular attachment, entry, replication, and translation (133).

However, Peli1 also plays a protective role against several viral infections. Peli1 participates in the positive feedback loop of IFN-β secretion by promoting IRF3 binding to the IFN promoter, which is crucial for IFN production during viral double-stranded RNA exposure (86). Peli1 also restricts herpes simplex virus type 1 (HSV-1) skin infection by suppressing HSV-1 replication and local dissemination. Peli1 enhances T cell recruitment to the infection by increasing Gpr156 expression (134). Peli1 positively regulates the antiviral response of isolated epithelial cells and the systemic response activated by TLR3. However, Peli1 plays an opposing role in the antiviral response in lung cells (78, 135). For example, Peli1 acts as a pro-inflammatory molecule during viral infection, and its knockdown reduces rhinovirus (RV)-induced CXCL8 and IL-6 production (78) without affecting RV replication (78, 135).

The role of Peli1 in infection depens on the infection type, which is common in studies on the role of other E3 ligases in infectious diseases. For example, TRIM29 exerts a pathogenic role by inhibiting I-IFN production during infections with Epstein–Barr and RNA viruses such as influenza and eutherian viruses (136–138). Peli1 positively regulates *Helicobacter pylori* and non-typeable *Haemophilus influenzae* (NTHi) infections (139, 140). Peli1 enhances TLR2-mediated NF-κB activity and chemokine-induced responses to H. pylori LPS (140). Inhibiting Peli1 improves bacterial clearance during NTHi infection (139). Thus, in infectious diseases, the differential targeting of Peli1 according to differences in its regulatory functions provides new pathways for treating infections.

### 5.4 Respiratory diseases

Peli1 is pathogenic in several respiratory diseases, notably Chronic obstructive pulmonary disease (COPD), asthma, acute lung injury, and persistent bacterial bronchitis (PBB). Peli1 primarily functions as a pro-inflammatory agent, influencing disease progression and severity. Peli1-mediated K63-Ub of P21 prevents P21 degradation, leading to an increased senescence-associated secretory phenotype and promotion of COPD and inflammation (42). Peli1 regulates the pro-inflammatory response of airway epithelial cells in patients with asthma, and Peli1 knockdown significantly reduces CXCL8 expression in the airway epithelium of these patients (78). E levation of IL-1 signaling factors, including Peli1, contributes to in childhood asthma. Elevaed Peli1 expression is associated with severity and relapse of asthma in patients (141).

Peli1 is also upregulated in acute lung injury and promotes disease progression (54, 90). TGF-β1 upregulates Peli1 expression in acute lung injury by suppressing microRNA-124 through DNA-methyltransferase 1 upregulation. This promotes M1 alveolar macrophage polarization via IRF5 nuclear translocation (54). Peli1 increases TRAF6 expression, activates the NF-κB pathway, and exerts pro-inflammatory effects (90). Furthermore, Peli1 expression is higher in children with persistent bacterial bronchitis (PBB), particularly in recurrent cases (142). In summary, Peli1 predominantly plays a pro-inflammatory role in COPD, asthma, acute lung injury, and PBB.

### 5.5 Neurological diseases

Alzheimer’s disease (AD) is a progressive neurodegenerative disorder characterized by β-amyloid (Aβ) accumulation in the brain (143, 144). Peli1 is a genetic risk factor for AD, and Peli1 expression is upregulated in the brain tissues of patients with AD and plays a pathogenic role (19, 61, 145). In AD, Peli1 mediates C/EBPβ ubiquitination degradation, which inhibits the CD36 expression, impairing Aβ phagocytosis by microglia (19). Peli1 has been implicated in AD pathogenesis through the TRAF3/MAPK and the BCL2 apoptotic pathways, thereby reducing microglial and neuronal cell viability. Peli1 downregulation through overexpression or mimicking MIR-590-5P, which is downregulated in AD, can attenuate neuronal damage caused by Aβ (61).

Stroke, one of the leading causes of death and disability, can benefit from accurate and rapid etiological classificationto determine treatment options and reduce the risk of recurrence. Peli1 is a diagnostic risk marker for Cardiogenic (CE) strokes (146–148). Elevated Peli1 levels are associated with a high risk of stroke in patients with atrial fibrillation, suggesting its potential use as a marker for stroke prediction, prevention, and treatment in this population (147).

Multiple sclerosis (MS) is a chronic inflammatory, demyelinating, and neurodegenerative disease of the central nervous system that affects young adults (149, 150). The role of Peli1 in Experimental autoimmune encephalomyelitis (EAE) pathogenesis is subject to contrasting viewpoints. Some studies have proposed that Peli1 positively regulates EAE progression by promoting microglial activation, whereas others suggest a protective role of Peli1 in EAE by inhibiting T-cell activation and pathogenicity. Peli1 promotes K48-Ub and degradation of TRAF3 by C-IAP (80), leading to MAPK activation, AP-1 activation, and inflammatory factors transcription, thus specifically activating microglia and promoting neuroinflammation. Conversely, Peli1 inhibits T-cell glycolysis, TH17 cell activation, and pathogenicity by mediating c-Rel ubiquitination degradation (13). Peli1-deficient EAE models have exhibited decreased inflammatory factors and EAE scores but elevated levels of peripheral autoimmunity and increased antigen-presenting proteins (151). Therefore, further investigations are required to comprehensively elucidate the specific mechanisms underlying the role of Peli1 in EAE, considering different cell types and their spatial and temporal contexts.

Peli1 is also involved in Methamphetamine (meth) abuse development, which leads to neurological symptoms, including memory impairment, altered cognitive function, and attention deficits (152). Peli1 plays a pro-inflammatory role in the effects of meth, as meth treatment results in elevated Peli1 expression owing to decreased levels of miR-142a-3p and miR-155-5p levels (56). Meth also upregulates Peli1 expression via the TRIF signaling pathway (99). Peli1 induces neuroinflammation by activating the NF-κB and MAPK pathways and RIPK1 (99, 153).

Peli1 is a potential therapeutic target for the treatment of neurological hemorrhagic diseases. Following subarachnoid hemorrhage (SAH), Peli1 upregulation induces MAPK activation and inflammatory factor production, activating microglia (94). Decreased miR-590-5p levels after intracranial hemorrhage lead to elevated Peli1 expression, inducing neuroinflammation and cerebral edema (62). Peli1 controls the survival of dopaminergic neurons by regulating microglia-mediated neuroinflammation and the production of neurotoxic factors through the NF-κB/MAPK pathway (154).

In summary, Peli1 is involved in the progression of various diseases within the nervous system via inflammation modulation, especially in the microglia. However, further research is required to elucidate the precise mechanisms and therapeutic implications of these effects.

### 5.6 Obstetrical diseases

Peli1acts as a pro-inflammatory pathogenic factor under various obstetric conditions. During ZIKV infection, Peli1 promotes ZIKV infection and placental inflammation, exacerbating congenital abnormalities (133). Peli1 is a novel regulator of TNF and TLR signaling in human myometrial and amniotic cells and upregulates the expression of pro-inflammatory factors, adhesion factors, and contractile proteinsPeli1 upregulation in the amniotic membranes of patients with preterm histological chorioamnionitis suggests its potential as a therapeutic target for reducing preterm delivery caused by inflammation and infection (155). I *in utero*, vitamin D intervention downregulate Peli1, contributing to immune protection against uterine inflammation (156). Peli1 positively correlates with miR-21 in patients with autoimmune premature ovarian insufficiency (POI). However, the underlying mechanisms and significance of Peli1 in POI pathogenesis require further investigation (157).

### 5.7 Autoimmune diseases

Peli1 regulates multiple autoimmune diseases with protective and pathogenic roles. Peli1 plays a pathogenic role in psoriasis (48, 158) and acts as an inflammatory modulatior in EAE, primarily promotes inflammation but inhibiting excessive inflammation (13, 80, 85). Systemic lupus erythematosus (SLE) is a complex, multisystem autoimmune disease characterized by genetic and environmental factors. In SLE, Peli1 is downregulated due to targeted inhibition by elevated miR-153-3p and miR-301a-3p levels (57, 60). Peli1 exerts its protective effects against SLE through multiple mechanisms. First, Peli1 downregulates the expression of inducible T cell co-stimulatory ICOS in CD4+ T cells by inhibiting c-Rel, inhibiting PI3K-AKT signaling. Upregulating downstream KLF2 inhibits Tfh and Th17 cell differentiation, ultimately attenuating autoimmunity in SLE (93). Second, Peli1 mediates the NIK ubiquitination and degradation, inhibits the nuclear translocation of Relb/P52, and prevents antibody production by B cells, thereby suppressing SLE (15). Peli1 inhibition of the non-classical NF-κB pathway in response to Poly IC treatment also attenuated SLE autoimmunity (159). Therefore, Peli1 is a promising protective factor against SLE and a potential therapeutic target.

Glucocorticoids are used extensively to treat inflammatory diseases. Glucocorticoid-induced GR downregulation is a well-known response that occurs in most cells and is necessary to limit the duration of glucocorticoid action (160). β-arrestin-1 binds to and remains stable at the GR (50). Silencing β-arrestin-1 results in the release of GR, leading to increased GR binding to the Peli1 promoter region. Subsequently, Peli1 transcription and expression are enhanced, and Peli1 mediates K48-linked ubiquitination of GR, contributing to GR turnover by shortening its half-life (50).

### 5.8 Sepsis

Sepsis, characterized by a systemic inflammatory response to infection, remains the leading cause of death in intensive care units (161). Pathological changes in sepsis are associated with the initial acute phase of hyperinflammation triggered by the innate immune system (162). Peli1 plays a prominent pro-inflammatory role in sepsis as an inflammatory factor. Peli1 promotes inflammation through TRAF6/NF-κB signaling and TRAF3/MAPK signaling (26, 90, 163). Peli1 couples the K63 Ub chain to ASC at the inflammatory vesicle junctions and promotes ASC/NLRP3 interactions and ASC oligomerization, leading to inflammatory vesicle activation (40). Peli1 induces TLR3- and TLR4-driven co-stimulatory gene expression, proliferation, and B cells survival (27). Peli1 deficiency enhances resistance to LPS endotoxic shock (40) and attenuates the induction of pro-inflammatory factors by TLR3/TLR4 ligands (27). In septic acute kidney injury (AKI) (64), DAPK1 Peli1 phosphorylation promotes RIP1 binding to caspase-8 and ultimately induces tubular apoptosis. In mice, synergistic DAPK1 inactivation or ablation and MyD88 inhibitors prevent septic AKI. In conclusion, Peli1 is implicated in sepsis and its progression in various organs and tissues through multiple pathways. Therefore, targeting Peli1 has a significant therapeutic potential for managing sepsis.

## 6 Potential of Peli1 as a therapeutic target

### 6.1 Inhibitors

The targeted regulation of Peli1 has emerged as an intriguing avenue for therapeutic intervention, given its diverse roles in disease pathogenesis. Several drugs have been identified as Peli1 inhibitors. For example, resistin inhibits Peli1 activity and suppresses invasive breast cancer metastasis. This inhibition is achieved through hydrogen bonding with specific Peli1 residues, inhibiting Peli1 enhanced SNAIL/SLUG activity via the K63 linkage (24) and slowing TNBC progression. BBT-401, a pharmacologically targeted Peli1 inhibitor developed by Bridge Biotherapeutics, is undergoing phase II clinical trials to treat ulcerative colitis. BBT-401-1S inhibits Th17 cell effector responses in psoriasis models by decreasing p65 phosphorylation and producing IL-17A. Peli1 inhibitors have also demonstrated a dose-dependent inhibition of IL-17A and IL-22 production (48).

### 6.2 Interaction blockers

Given that Peli1 often functions through protein interactions, the regulation of Peli1 binding to target proteins and co-targeting strategies have been explored. Peli1 and EGFR promote metastasis in breast cancer. Compound S62, which does not individually bind to Peli1 or EGFR, can block both linkages and shows promise for treating breast cancer with combined targeting of Peli1 and EGFR (22). Furthermore, Peli1 has been implicated in modulating the tolerance to JQ1 targeting BRD4 in breast cancer. Combining BRD4 and Peli1 targeting is necessary for effective breast cancer treatment (21). Peli1 expression is increased in breast cancer samples from patients receiving multiple chemotherapeutic agents, and its expression level is positively correlated with the number of agents used by patients (21). This finding indicates that Peli1 is involved in resistance to various agents, warranting further investigation (21). A novel membrane-bound palmitate-coupled Smad6-derived peptide called Smaducin-6 has been developed to disrupt IRAK1-, RIP1-, and Ikkϵ-mediated TLR4 signaling complex formation by interacting with membrane-bound Peli1 and to restore neutrophil recruitment by reducing GRK2 expression in neutrophils through CXCR2 re-expression is sepsis (87).

### 6.3 Gene therapy

Gene therapy targeting Peli1 has also shown promise in improving perfusion and cardiac function in ischemic infarction models (95, 105). In an LPS-induced myocarditis model, Peli1 activatio was associated with promoting pro-inflammatory genes, and si-Peli1 treatment alleviated or reversed LPS-induced cellular injury by altering cardiomyocyte energy metabolism (129). MiRNAs have been explored as regulators of Peli1 and human umbilical cord mesenchymal stem cell-derived extracellular vesicles modified with miR-30c-5p effectively suppress Peli1 expression and inhibit papillary thyroid carcinoma progression *in vitro* and *in vivo* (103).

### 6.4 Other drug developments

Targeted protein degradation (TPD) and targeted covalent inhibitors (TCIs) may provide insights into the targeting Peli1 (164, 165). TPD primarily comprises molecular glue and proteolysis targeting chimera (PROTAC), which mediate the binding of E3 Ub ligases to target proteins, leading to target proteins degradation. PROTACs are heterobifunctional molecules comprising two specific ligands and a chemical linker that enables them to bind to E3 Ub ligases and the target protein (166). For example, von Hippel-Lindau (VHL) and cereblon (CRBN) are still the most widely used E3 ligases, and many of the developed PROTACs are based on their efficacy (167–169). Molecular glues are low- molecular- weight inducers or protein-protein interaction stabilizers.Upon binding to a protein, the small molecule induces a conformational change and causes the small molecule-protein complex to become a “new substrate” for the E3 ligase and thus undergoes ubiquitination. For example, thalidomide and its derivatives are effective cancer therapeutic agents and are among the best understood molecular glue degraders (11). These drugs selectively reprogram the E3 ligase cereblon (CRBN) to allow the Ub-proteasome system to degrade target proteins. In addition to promoting Peli1 degradation as a target protein through TPD, inhibiting Peli1 function through covalent inhibitors is also a potent and effective strategy. Covalent inhibitors are a class of small molecule compounds that can covalently bind to specific target proteins, inhibiting their biological functions, similar to the study of BBT-401 inhibiting Peli1 (48). However, Peli1 agonists are not being developed, which is detrimental to studies targeting the protective role of Peli1 in diseases such as SLE. Recently, with advances in computational tools, structure-targeted, high-throughput virtual screening and molecular docking have become effective methods for inverse drug discovery. TCI and agonist by high-throughput virtual screening of Peli1 may be helpful in targeted therapy.

### 6.5 Potential side effects

Targeting Peli1 and therapy via TPD-linked Peli- targeting of downstream substrates may have side effects. Due to the diversity of Peli1 substrates and the wide range of biological functions in which Peli1 is involved, we also noted that Peli1 is primarily pathogenic in some diseases such as cancer, myocardial infarction, and AD. In contrast, Peli1 plays a protective role in other diseases such as atherosclerosis, some infectious diseases, and SLE. Complex roles make targeting Peli1 challenging, and Peli1 inhibition or promotion should be discussed according to the disease context, especially when patients simultaneously have different diseases in which Peli1 is involved.

Peli1 is critical in the development and progression of various diseases, particularly immune-related disorders and cancers. Further research on Peli1 as a diagnostic and therapeutic target, aided by cutting-edge therapeutic tools, may provide novel treatment options for various of human diseases. T he side effects of therapeutic interventions on Peli1 in different contexts in response to the complex regulation of Peli1 are also worth studying.

## 7 Discussion

As a significant member of the E3 ligase family Peli, Peli1 exerts its regulatory function by binding to various proteins and mediating post-translational ubiquitination modifications (7, 8). Peli1 also exhibits multiple functions independent of its E3-Ub ligase activity, expanding its regulatory repertoire (35, 43, 47). The reversible phosphorylation mechanism enables the interconversion of Peli1 between inactive and active forms, with various kinases (such as IRAK1, IRAK4, TBK1/Ikkϵ) phosphorylating Peli1 to enhance its E3 ligase activity (36, 63). Peli1 possesses numerous phosphorylation sites, rendering it prone to activation through kinase phosphorylation and less susceptible to dephosphorylation-mediated inactivation (22, 85). Peli1 production and degradation may represent crucial pathways for its negative regulation. Peli1 transcription is regulated by IRF3 and the GR (50). Several miRNAs have been identified as Peli1 regulators via direct targeting (53–55, 60). Peli1 can also promote miR-21-mediated negative regulation of its expression through NF-κB (51), suggesting a potential negative feedback loop. IRAK-1 facilitates the Ub proteasome-dependent Peli1 degradation in a kinase-dependent manner (7); however, the specific mechanism of Peli1 degradation remains unknown.

Peli1 participates in the signaling through receptor systems such as IL-1R and TLRs (27, 72, 80). Peli1 exerts a multifunctional regulatory role in signaling pathways, including NF-κB, MAPK, and AKT (90, 93, 97), which are involved in pro-inflammatory responses and immune progression. Paradoxically, Peli1 also inhibits T cell activation by suppressing excessive NF-κB activation (13). Peli1 is involved in cell death, autophagy, DNA repair, glycolysis, and immune cell activation. The reasons underlying the paradoxical role of Peli1 in inflammatory regulation remain unknown, and a deeper understanding of its biology will contribute to our understanding of innate and adaptive immunity.

Peli1 has distinct roles in the progression of various diseases within different disease contexts. Peli1 acts as a pathogenic agent in most tumors (23, 24, 103), where it promotes tumor cell migration and proliferation and negatively affects antitumor immunity. However, in some tumor backgrounds, Peli1 is a beneficial factor with a protective function in antitumor immunity (37), and promotes radiation therapy sensitivity in esophageal cancer (16). Peli1 is a crucial regulatory molecule of the immune system in the cardiovascular system, exerting pro-inflammatory and pro-apoptotic effects (92). Given its specific expression in microglial, Peli1 functions in progression of various CNS diseases (e.g., AD, cardiogenic stroke, and MS) by regulating microglia (19, 80, 146). Peli1 promotes neuroinflammation after treatment with central nervous system drugs such as meth and morphine. Peli1 also differentially regulates several autoimmune diseases. Although its pathogenic role in MS and psoriasis is well- recognized, Peli1 plays a protective role against SLE. During viral infections, Peli1 plays a pro-inflammatory role (38, 88, 89, 133) and contributes to the pathogenesis of viral infections such as VSV, WNV, HIV, and ZIKV by promoting viral replication. However, Peli1 protects against HSV infection (134) by limiting viral invasion and spread. In contrast, Peli1 downregulation in JEV infection promotes immune evasion (59).

Thus, the significance of targeting Peli1 and its downstream target genes is evident. BBT-401 (48), a Peli1 target, is undergoing phase II clinical trials to treat ulcerative colitis. Several other drugs have shown promise in targeting Peli1, including the Smad6-derived peptide Smaducin-6, compound S62, and resistin (22, 24, 133). Blocking Peli1 can confer protective effects in numerous diseases; however, exploring Peli1 agonists remains an intriguing area of investigation, as they could potentially enhance the protective effects of Peli1 in diseases such as SLE. TPDs and TCIs have been used as cutting-edge therapeutic strategies based on several E3 ligases, providing new directions for developing of targeted therapies for Peli1. However, side effects of targeting Peli1 are inevitable, considering the involvement of Peli1 in multiple diseases and biological functions. Therefore, novel drug development and individualized therapeutic strategies are needed to target Peli1 within reasonable limits and avoid adverse events as much as possible.

Mounting evidence supports the predictive and prognostic value of Peli1. Therefore, a comprehensive understanding of the biological properties and molecular functions of Peli1 will facilitate the development of novel clinical therapeutic strategies. This review will advance future research on the role of Peli1 in immune diseases, cancers, and other conditions. This review provides information on the molecular mechanisms and directions for the clinical diagnosis and treatment of diseases, offering fundamental insights and evidence for potential future research areas, particularly in inflammation and cancer.

## Author contributions

LY: Visualization, Writing – original draft, Writing – review & editing. YC: Investigation, Resources, Writing – review & editing. JF: Writing – review & editing, Supervision.
